# Population Genomics Provide Insights into the Global Genetic Structure of *Colletotrichum graminicola*, the Causal Agent of Maize Anthracnose

**DOI:** 10.1128/mbio.02878-22

**Published:** 2022-12-19

**Authors:** Flávia Rogério, Riccardo Baroncelli, Francisco Borja Cuevas-Fernández, Sioly Becerra, JoAnne Crouch, Wagner Bettiol, M. Andrea Azcárate-Peril, Martha Malapi-Wight, Veronique Ortega, Javier Betran, Albert Tenuta, José S. Dambolena, Paul D. Esker, Pedro Revilla, Tamra A. Jackson-Ziems, Jürg Hiltbrunner, Gary Munkvold, Ivica Buhiniček, José L. Vicente-Villardón, Serenella A. Sukno, Michael R. Thon

**Affiliations:** a Instituto de Investigación en Agrobiotecnología (CIALE), Departamento de Microbiología y Genética, Universidad de Salamanca, Salamanca, Spain; b Department of Agricultural and Food Sciences (DISTAL), University of Bologna, Bologna, Italy; c Foreign Disease and Weed Science Unit, United States Department of Agriculture, Fort Detrick, Maryland, USA; d Embrapa Environment, Jaguariúna, São Paulo, Brazil; e Center for Gastrointestinal Biology and Disease, Department of Medicine, School of Medicine, University of North Carolina, Chapel Hill, North Carolina, USA; f Division of Gastroenterology and Hepatology, Department of Medicine, School of Medicine, University of North Carolina, Chapel Hill, North Carolina, USA; g UNC Microbiome Core, Department of Medicine, School of Medicine, University of North Carolina, Chapel Hill, North Carolina, USA; h USDA Animal and Plant Health Inspection Services, Biotechnology Regulatory Services, Riverdale, Maryland, USA; i Syngenta Seeds La Grangette, Lombez, France; j Bayer Crop Science/Monsanto SAS, Monbequi, France; k Ontario Ministry of Agriculture, Food, and Rural Affairs, University of Guelph-Ridgetown, Ridgetown, Ontario, Canada; l Facultad de Ciencias Exactas Físicas y Naturales, Universidad Nacional de Córdoba, IMBIV-CONICET-ICTA, Córdoba, Argentina; m Department of Plant Pathology and Environmental Microbiology, The Pennsylvania State University, State College, Pennsylvania, USA; n Misión Biológica de Galicia, Spanish National Research Council (CSIC), Pontevedra, Spain; o Department of Plant Pathology, University of Nebraska-Lincoln, Nebraska, USA; p Federal Department of Economic Affairs, Zurich, Switzerland; q Department of Plant Pathology and Microbiology, Iowa State University, Ames, Iowa, USA; r BC Institute for Breeding and Production of Field Crops, Dugo Selo, Croatia; s Statistics Department, University of Salamanca, Salamanca, Spain; Universidad de Córdoba

**Keywords:** fungal plant pathogen, population genetics, phylogeography, phytopathology, recombination, migration, population genomics

## Abstract

Understanding the genetic diversity and mechanisms underlying genetic variation in pathogen populations is crucial to the development of effective control strategies. We investigated the genetic diversity and reproductive biology of Colletotrichum graminicola isolates which infect maize by sequencing the genomes of 108 isolates collected from 14 countries using restriction site-associated DNA sequencing (RAD-seq) and whole-genome sequencing (WGS). Clustering analyses based on single-nucleotide polymorphisms revealed three genetic groups delimited by continental origin, compatible with short-dispersal of the pathogen and geographic subdivision. Intra- and intercontinental migration was observed between Europe and South America, likely associated with the movement of contaminated germplasm. Low clonality, evidence of genetic recombination, and high phenotypic diversity were detected. We show evidence that, although it is rare (possibly due to losses of sexual reproduction- and meiosis-associated genes) *C. graminicola* can undergo sexual recombination. Our results support the hypotheses that intra- and intercontinental pathogen migration and genetic recombination have great impacts on the *C. graminicola* population structure.

## INTRODUCTION

Crop diseases are one of the biggest concerns in global agriculture, responsible for major losses in food production (1, 2). Fungi are the primary causal agents of plant diseases and can cause catastrophic damage (3, 4). Disease outbreaks caused by fungal plant pathogens have increased in recent decades and are recognized as emergent threats to food security worldwide (5–8). Human activity tends to intensify fungal disease dispersal by promoting the movement of pathogens far from their native location (9, 10). The modification of natural environments caused by modern agriculture provides new opportunities for natural selection (10). Employing crops with limited genetic diversity increases the risk of global disease spread because pathogen genotypes can rapidly spread through genetically uniform host populations (11, 12). Additionally, ongoing climate changes can shift plant-pathogen distributions on a global scale because temperature changes may affect disease development, aiding in the spread and survival of pathogens and their establishment in hitherto unsuitable regions (8, 12, 13).

Maize (Zea mays) is the second most extensively cultivated cereal crop globally, with a harvested area of almost 200 million hectares (14). Together with rice and wheat, maize supplies nearly half of all daily calories in human food, with maize being responsible for roughly 10% of that total (14, 15). Although many diseases negatively impact maize production, anthracnose is one of the most important diseases, and requires the use of integrated practices to control it (16–19). Anthracnose disease is caused by *Colletotrichum graminicola*, a haploid ascomycete fungus that can infect most plant tissues, although stalk rot and seedling blight can cause the most significant economic damage (20–22). Taxonomically, *C. graminicola* belongs to the Graminicola species complex, a well-defined monophyletic clade encompassing 16 *Colletotrichum* species pathogenic to the Poaceae family (22–24).

Prior to 1960, maize anthracnose was a minor leaf spot disease in the USA, and the causal agent *C. graminicola* was primarily studied for its taxonomic significance (21–25). However, this view changed during the following decades, when devastating outbreaks caused unexpected losses across the north-central and eastern regions of the USA (18, 21). Afterwards, the disease became increasingly common and, in some seasons, extremely destructive throughout the eastern USA (26, 27). The occurrence of these outbreaks is thought to be associated with the use of hybrids which are particularly susceptible to anthracnose, more favorable weather conditions, and the emergence of more aggressive strains of the pathogen with its rapid spread through the maize belt (28–30). Nowadays, maize anthracnose is a well-established endemic disease in the USA, and it is better managed, mainly through the use of resistant cultivars, but it still can cause important damage in some regions of the country (31–35). Given the history of the disease, it may have the potential to become much more significant in the future.

Outside the USA, *C. graminicola* is globally distributed, and it is of major importance in the Americas and other regions of the world (36). In North America, Canada reports variable disease incidence, but anthracnose is still a significant concern (34, 37, 38). In addition to temperate areas, maize anthracnose is also reported in tropical and subtropical regions, including Brazil and Argentina, where warm and humid conditions contribute to augment the disease (39–46). Since the first report of maize anthracnose in Europe (Italy in 1852) (47), several countries have reported its occurrence, including Germany, France, the former Czechoslovakia (48–50), and more recently Croatia, Portugal, Switzerland, Bosnia, and Herzegovina (51–54). Recently, maize anthracnose was also reported in China (55), indicating that the pathogen’s geographic range is increasing.

The wide dissemination of pathogens over continents indicates an intense dispersal of their propagules, either from aerial dispersal or via human-mediated movement of infected plant material and/or seeds (11, 56, 57). The nature of fungal propagules, i.e., their small and numerous spores, favors long-distance dispersal, resulting in the spread of pathogens across and between continents (11). Although many pathogens are cosmopolitan in their distribution, their populations often differ in some respects: for instance, virulence, resistance to chemical controls, and genetic diversity (58). The migration of plant pathogens has epidemiological and evolutionary implications because the movement of individuals from one population to another may result in gene flow when migrants contribute their genes to the gene pool of another population (59). *Colletotrichum graminicola* is seed-transmitted, also known as a seed-born pathogen (60–62); therefore, contaminated seeds serve as inoculum sources and promote pathogen movement. Furthermore, through the transport of seeds and infected plant material, fungi can naturally be spread via the dispersal of both sexual and asexual spores (11, 56, 63). In addition to the dispersal process, the reproductive biology of a species is crucial to understanding the evolutionary forces which shape the genetic diversity of pathogen populations across time and space (64). Asexual reproduction predominates in most plant-pathogenic fungi, but many species undergo sexual cycles, and many others employ both reproductive strategies (59). Sexual reproduction produces genetic recombination, increasing genotype diversity and generating random associations between alleles at different loci (65, 66). However, alternative mechanisms, such as parasexual reproduction and a cryptic sexual cycle, can also create genetic recombination in fungal species (67, 68). Although sexual reproduction may be costly (in terms of fitness) and disrupt favorable gene combinations, experimental evidence has suggested that sexual reproduction in fungi may increase the rate of adaptation to new environments (69, 70). Recombination can create particular genotypes through the combination of more fit alleles, which can accelerate the evolution of strains which have resistance genes against multiple fungicides and overcome the R genes of resistant cultivars (65, 71, 72).

The genetic control underlying sexual development in the genus *Colletotrichum* is not well known, and it differs from the typical bipolar mating system of ascomycetes (73–75). All species studied so far contain just one of the two mating types alleles typical of ascomycetes (*MAT1-1/2*) (22), which would seemingly limit their ability to mate. However, sexual reproduction has been demonstrated for at least some *Colletotrichum* species, including *C. graminicola*. Although the teleomorph of *C. graminicola* has been characterized under laboratory conditions (74, 75), the asexual state is more often found in the field and plays a significant role in the life cycle. This species is reported to be homothallic (74), i.e., possessing both sets of genes, and thus can complete a sexual cycle independently (76). However, some strains are self-sterile and cross-fertile (75, 77), which means that some strains can reproduce sexually when cultured alone while others require a mating partner to produce sexual offspring.

Genetic recombination is reported to be an important factor in the ecology and evolution of many *Colletotrichum* species (78–83). However, only a few population studies have characterized the genetic diversity of grass-infecting *Colletotrichum* species, such as *C. sublineola* and *C. cereale* (81–85), and little is known about *C. graminicola* population diversity. Studies using single marker genes and limited sample sizes have shown high genetic variability in *C. graminicola* isolates (83, 86). Therefore, in this study, we investigated the global population structure of *C. graminicola* to gain insights into its reproductive biology. Our population genomic analyses using single-nucleotide polymorphisms (SNPs) revealed population subdivision into three genetic clades with isolates grouped according to their country of origin, with distinct levels of genetic diversity between them, suggesting different evolutionary histories. We found that intra- and intercontinental migration, mainly between Europe and South America, is likely associated with the movement of contaminated seeds. We discussed the possible evolutionary origins of these genetic groups, the impact of recombination on the genetic structure, and the potential for maize anthracnose to become a much more significant disease in the future.

## RESULTS

### Population genetic analyses reveal three lineages of *C. graminicola*.

**FIG 1:**
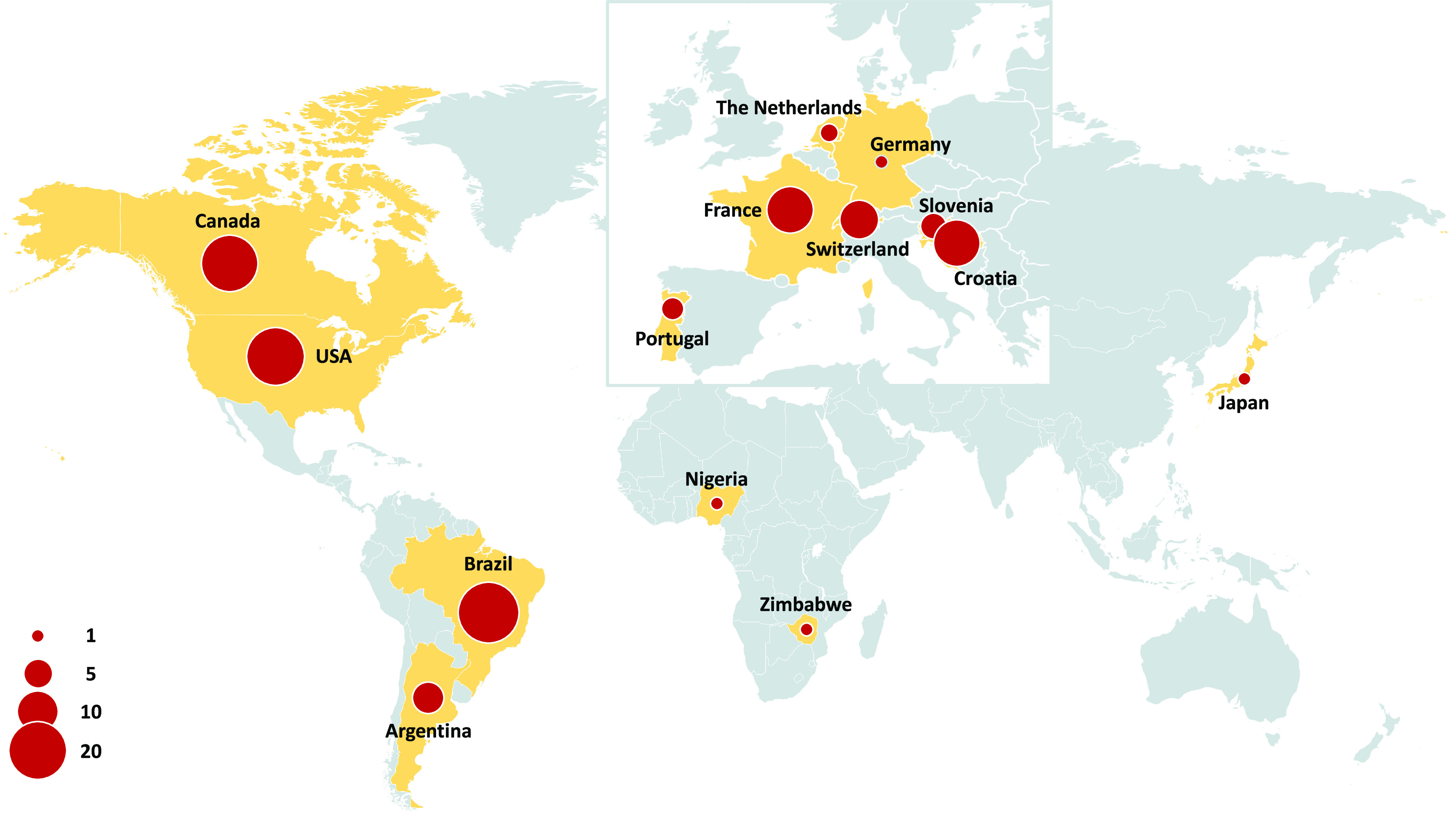
Worldwide *Colletotrichum graminicola* sampling. Red circles indicate the number of isolates sampled. Artwork created with mapchart.net.

A total of 5,811 biallelic variants were detected among 108 *C. graminicola* isolates collected from 14 countries, and these were used for population genetic analyses (Fig. 1, Table S1). Analysis of SNP density showed an average of 1.4 SNPs per 10 kb across all contigs. Information on reads and coverage per individual is provided in Table S1 in the supplemental material.

10.1128/mbio.02878-22.5 TABLE S1 Isolates of *Colletotrichum graminicola* used in this study. Download Table S1, XLSX file, 0.04 MB. Copyright © 2022 Rogério et al. 2022 Rogério et al. https://creativecommons.org/licenses/by/4.0/ This content is distributed under the terms of the Creative Commons Attribution 4.0 International license.

**FIG 2:**
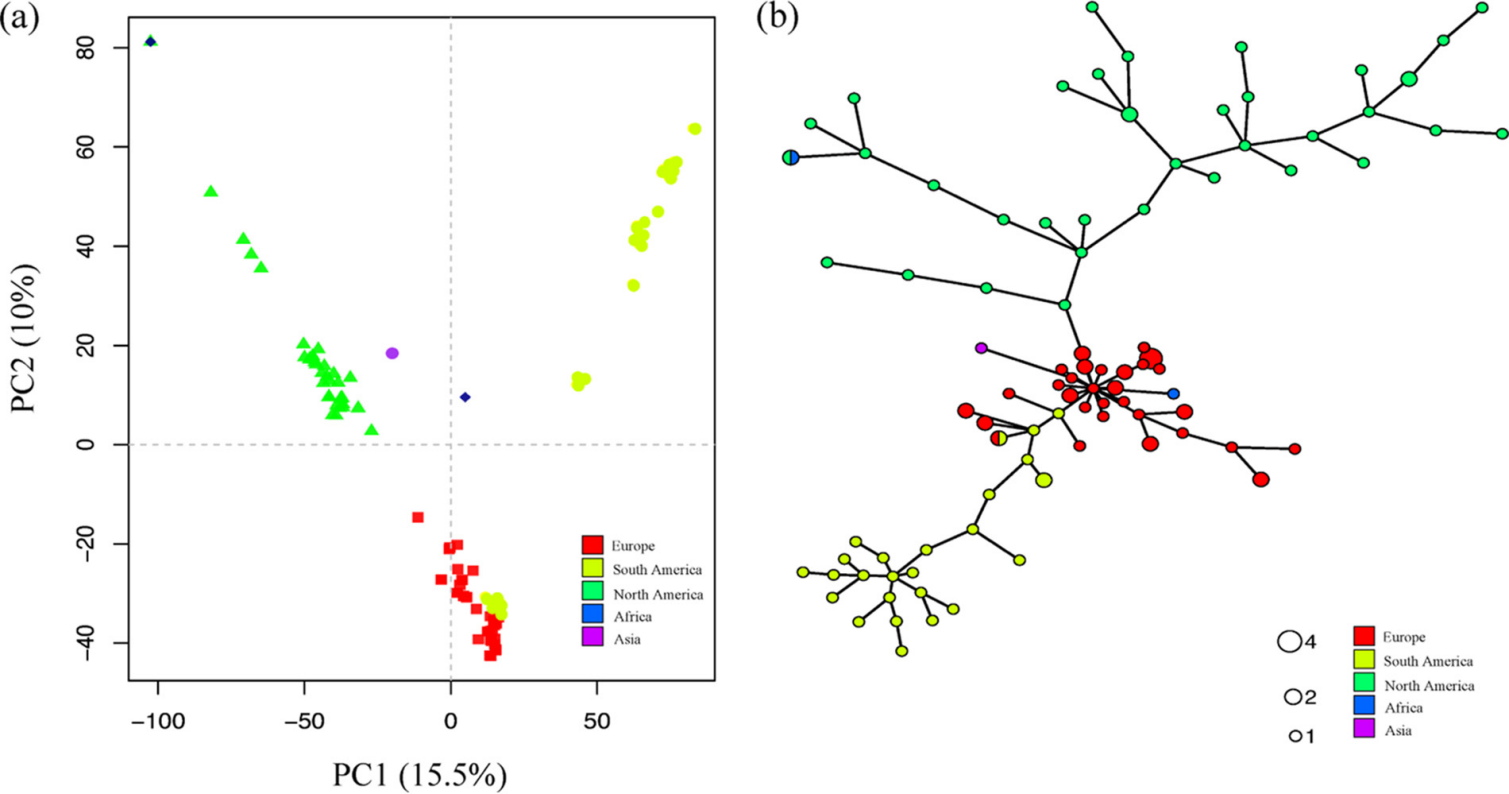
Population subdivision of *Colletotrichum graminicola*. (A) Principal-component analysis (PCA). Isolates are colored according to their continent of origin. (B) Minimum spanning network (MSN) showing the multilocus genotypes (MLGs) based on Nei’s genetic distance. The distance between nodes represents the genetic distance between MLGs. Each circle represents a distinct MLG, proportional in size to the number of isolates with the genotype.

Clustering methods revealed population subdivisions, supporting the existence of three genetic groups. A principal-component analysis (PCA) and discriminate analysis of principal components (DAPC) grouped individuals according to their geographic origin (Fig. 2a, Fig. S1). However, all isolates from Argentina grouped together with the European isolates, suggesting migration between groups. The isolates CBS113173 and MAFF511343, from Zimbabwe and Japan, respectively, and obtained from culture collections did not group with the main three genetic clusters, suggesting that they represent additional major *C. graminicola* clades. However, larger samples from these geographic regions would be necessary to draw any conclusions.

10.1128/mbio.02878-22.1 FIG S1 (A) Bayesian information criteria (BIC) indicating the most likely number of genetic groups by discriminant analysis of principal component analysis (DAPC). (B) Scatterplot from DAPC of *Colletotrichum graminicola* isolates assigned with *a priori* geographical information (by continent). Download FIG S1, PDF file, 0.1 MB. Copyright © 2022 Rogério et al. 2022 Rogério et al. https://creativecommons.org/licenses/by/4.0/ This content is distributed under the terms of the Creative Commons Attribution 4.0 International license.

**FIG 3:**
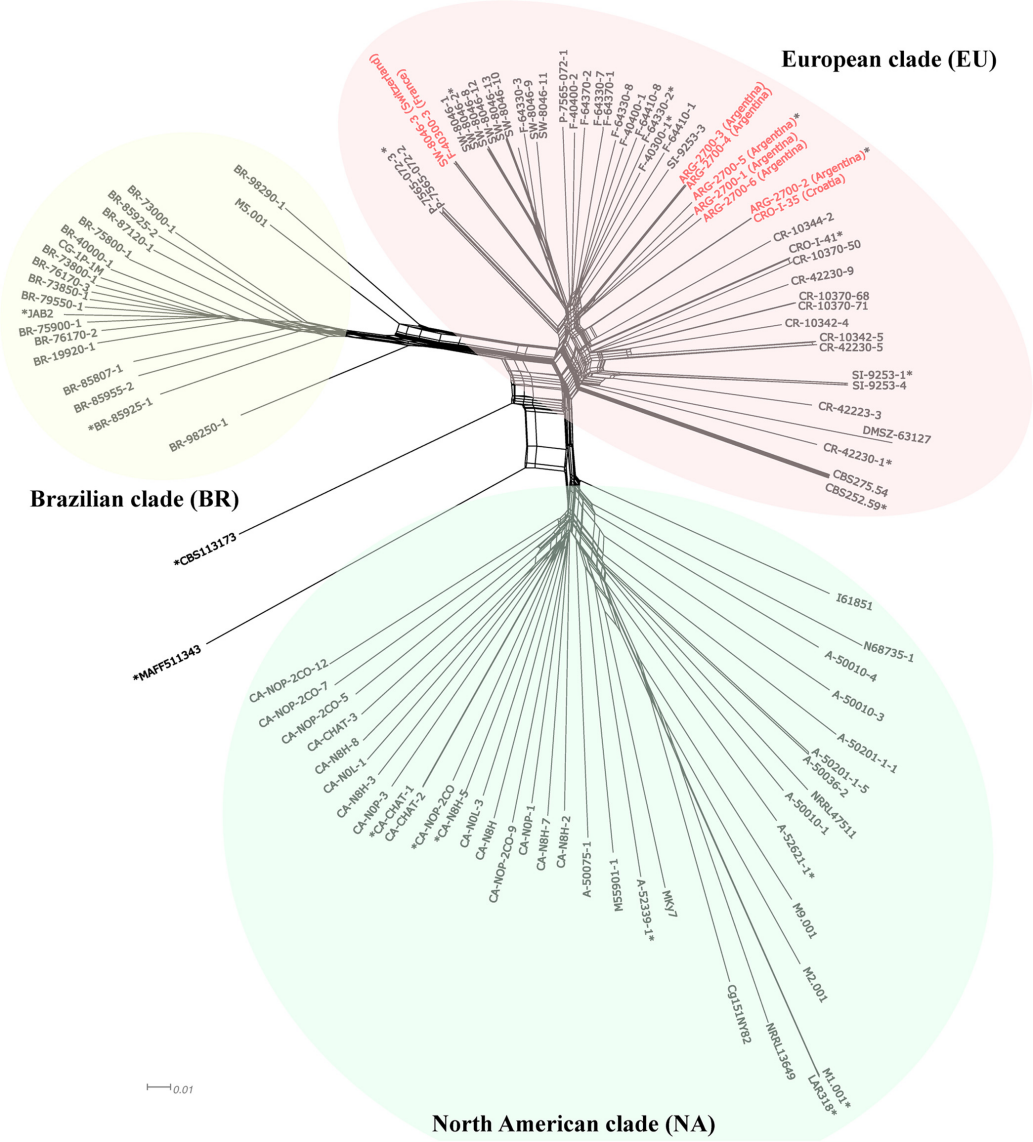
Neighbor-net network showing relationships between *Colletotrichum graminicola* isolates. Red color indicates “migrant” isolates, i.e., isolates in the European clade. Isolates with pathogenic characterization are indicated by asterisks.

The snapclust function, based on the Bayesian information criterion (BIC) criterion, supported the clustering pattern, showing an optimum value of *K *=* *3 clusters (Fig. S2). A neighbor-net network generated by SplitsTree also identified three groups in the data, here referred to as the Brazilian (BR), European (EU), and North American (NA) clades (Fig. 3). Interestingly, some isolates clustered with clusters of isolates from other geographic locations, as indicated in Fig. 3 (red). An analysis of molecular variance (AMOVA) performed between *C. graminicola* clusters showed that 65.5% of the total genetic variance was within clusters and 34.5% was among them. Pairwise *F*_ST_ values between clusters were significant and revealed strong differentiation, ranging from 0.26 to 0.42 (Table S3).

10.1128/mbio.02878-22.2 FIG S2 Bayesian information criteria (BIC) indicating the most probable number of genetic groups using the snapclust function. Download FIG S2, PDF file, 0.1 MB. Copyright © 2022 Rogério et al. 2022 Rogério et al. https://creativecommons.org/licenses/by/4.0/ This content is distributed under the terms of the Creative Commons Attribution 4.0 International license.

10.1128/mbio.02878-22.7 TABLE S3 Pairwise genetic differentiation between genetic clusters of *Colletotrichum graminicola.* Download Table S3, XLSX file, 0.1 MB. Copyright © 2022 Rogério et al. 2022 Rogério et al. https://creativecommons.org/licenses/by/4.0/ This content is distributed under the terms of the Creative Commons Attribution 4.0 International license.

Among the 108 isolates, we detected 76 unique multilocus genotypes (MLGs) and 15 MLGs shared between at least 2 isolates. Most MLGs included isolates from the European clade (13 MLGs in total) and were shared between isolates from the same clade and country (Table S4). However, one MLG encompassed isolates from France and Switzerland (F-40300-3 and SW-8046-3), and another encompassed isolates from Argentina and Croatia (ARG-2700-2 and CRO-I-35). Two isolates from culture collections (M1.001 and LAR318) ins the USA and Nigeria were also shared. These shared genotypes were almost identical genetically, with few SNPs between samples (Table S4). The minimum spanning network (MSN) showed a similar relationship among these genotypes. The European cluster displayed an inner position with genetically closer MLGs, while the Brazilian and North American clusters were placed on the edges of the network, likely due to their separate evolutionary histories.

10.1128/mbio.02878-22.8 TABLE S4 Multilocus genotypes (MLGs) among *Colletotrichum graminicola* isolates. Download Table S4, XLSX file, 0.01 MB. Copyright © 2022 Rogério et al. 2022 Rogério et al. https://creativecommons.org/licenses/by/4.0/ This content is distributed under the terms of the Creative Commons Attribution 4.0 International license.

To estimate genetic diversity, we computed the number of segregating sites within genetic groups. Genetic diversity was twice as high in the North American clade compared to the Brazilian and European clades (Table 1). Based on all the SNPs from the full data set, the North American clade showed almost all sites segregating when all isolates belonging to this clade were considered. In contrast, isolates from the European clade showed fewer segregating SNPs and consequently lower genetic diversity.

**TABLE 1:** SNP variation within *Colletotrichum graminicola* clusters^*a*^

Cluster	SNPs (*n*)
BR	2,484
EU	2,077
NA	5,264

a SNP, single-nucleotide polymorphism; BR, Brazil; EU, Europe; NA, North America.

### Footprints of recombination.

**FIG 4:**
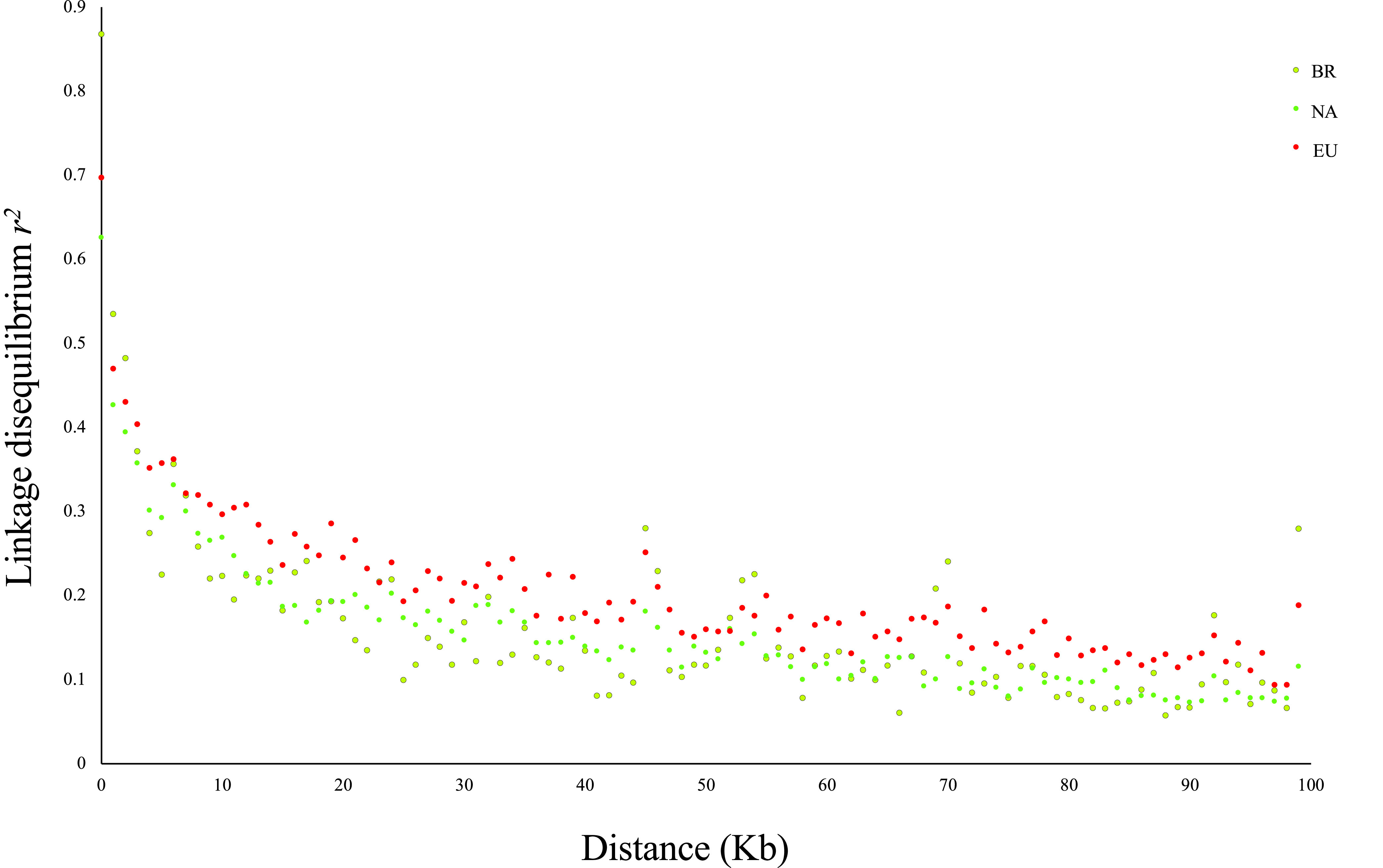
Linkage disequilibrium (LD) decay plots of three genetic populations of *Colletotrichum graminicola* (Brazilian, North American, and European clades). LD was calculated for all pairs of SNPs less than 100 kb apart from each other.

We assessed the genomic impact of recombination by analyzing patterns of linkage disequilibrium (LD). For this, we computed LD on the genetic clusters separately to avoid combining evolutionarily distinct populations, which can lead to overestimation of the linkage equilibrium (65). For all genetic clusters, the LD decayed quickly with physical distance (Fig. 4), reaching 50% of its maximum value in less than 10 kb; this suggests a random association of alleles at different loci, likely caused by recombination events. We also used the pairwise homoplasy index (PHI) test implemented in SplitsTree to test the null hypothesis of no recombination, which rejected the null hypothesis for all three genetic populations (*P* < 0.001).

To test the hypothesis that *C. graminicola* sexual reproduction- and meiosis-associated genes were maintained, we explored the presence of 96 genes previously identified as having roles in sexual reproduction in Ascomycetes. We observed the presence of 91 out of 96 sex-related genes in three *C. graminicola* genomes (Table S5). Five genes, *HOP1*, *HOP2*, *MND1*, *PRE1*, and *PRE2*, were not found in the *C. graminicola* genomes. The genes *HOP1*, *HOP2*, and *MND1* were also not found in the sexually reproducing fungi Neurospora crassa, Podospora anserina, and *Nectria haematococca*, nor in any of the Sordariomycetes, which may indicate that they are not required for the sexual cycle in this group (87). The genes *PRE1* and *PRE2* are also not essential for mating, as no phenotypic change was observed with gene-deletion mutants of these genes in *C. fructicola* (88). Only the isolates M1.001, LARS318, and CBS113173 maintained all 91 genes needed for sexual reproduction.

10.1128/mbio.02878-22.9 TABLE S5 *Colletotrichum graminicola* orthologues of sex-related genes described in Neurospora crassa, Saccharomyces cerevisiae, Podospora anserina, Fusarium graminearum, and Schizosaccharomyces pombe. Download Table S5, XLSX file, 0.1 MB. Copyright © 2022 Rogério et al. 2022 Rogério et al. https://creativecommons.org/licenses/by/4.0/ This content is distributed under the terms of the Creative Commons Attribution 4.0 International license.

### Pathogenicity assays reveal large variation in virulence.

**FIG 5:**
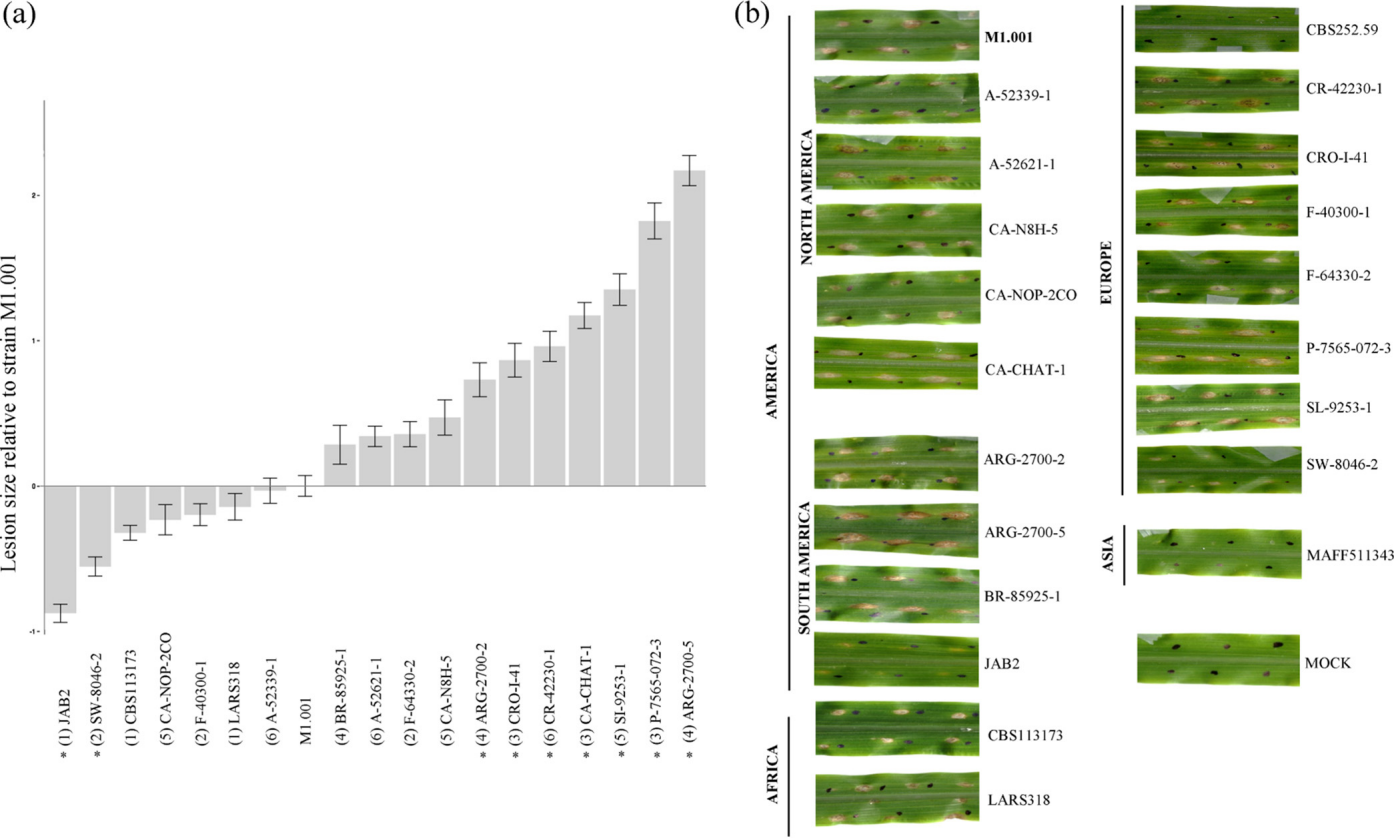
Pathogenic characterization of *Colletotrichum graminicola* isolates. (a) Graphic representation of 19 isolates in order of virulence (relative to the control strain M1.001) in ascending order. The isolates CBS252.59 and MAFF511343 were nonpathogenic to the maize cultivar evaluated and are not shown in the figure. Pathogenicity assay batch number is shown in parentheses. Isolates marked with an asterisk were significantly different (*P* < 0.001) from strain M1.001 based on a *post hoc* Tukey’s test using a Bonferroni correction. (b) Necrotic lesions on maize leaves at 4 days after inoculation with spore suspensions of the isolates.

Twenty-one isolates representative of the clades identified in the phylogeny inference and showing diverse colony morphology and pigmentation were chosen for pathogenic characterization (Fig. S3). Considering their continental origins, we chose the following isolates: America, M1.001, A-52339-1, A-52621-1, CA-N8H-5, CA-NOP-2CO, CA-CHAT-1, ARG-2700-2, ARG-2700-5, BR-85925-1, and JAB2; Africa, CBS113173, LARS318; Europe, CBS252.59, CR-42230-1, CRO-I-41, F-40300-1, F-64330-2, P-7565-072-3, SI-9253-1, and SW-8046-2; and Asia, MAFF511343. All isolates except two, CBS252.59 and MAFF511343, were pathogenic on the maize cultivar evaluated, producing necrotic lesions at the inoculation points by 4 days postinoculation (dpi) (Fig. 5). Isolates CBS252.59 and MAFF511343 also failed to produce necrotic lesions at 6 dpi (data not shown). An analysis of variance (ANOVA) revealed significant differences in virulence among the isolates (*P* < 0.0001) and a *post hoc* Tukey’s test revealed seven isolates which were significantly more virulent and two which were significantly less virulent than M1.001 (Fig. 5). Population structure was not correlated with virulence.

10.1128/mbio.02878-22.3 FIG S3 Colony morphology of *Colletotrichum graminicola* showing phenotypic diversity among isolates. The colonies were cultivated on PDA medium, at 23°C under continuous light for 6 days. Download FIG S3, PDF file, 1.3 MB. Copyright © 2022 Rogério et al. 2022 Rogério et al. https://creativecommons.org/licenses/by/4.0/ This content is distributed under the terms of the Creative Commons Attribution 4.0 International license.

## DISCUSSION

We used a population genomics approach to determine the genetic diversity of *C. graminicola* isolates infecting maize sampled from 14 countries. Using SNP markers generated through restriction site-associated DNA sequencing (RAD-seq) and whole-genome sequencing (WGS), we investigated the distribution of genetic variation considering our samples as a global population. We found populational differentiation on a global scale, with three genetic groups delimited by continental origin, consistent with short dispersal of the pathogen and geographic subdivision. However, we observed intra- and intercontinental migration, possibly associated with the movement of contaminated seeds. The genetic clusters, corresponding to the Brazilian, European, and North American clades, showed distinct levels of genetic diversity which could reflect different evolutionary histories. Analysis of linkage disequilibrium and the pairwise homoplasy index (PHI) test for clonality provided evidence of genetic recombination. Investigation of sex-related genes revealed gene losses in most isolates. We have shown evidence that, although it is rare (possibly due to losses of sexual reproduction- and meiosis-associated genes), *C. graminicola* can undergo sexual recombination based on lab assays and genomic analyses. We present the possible evolutionary origins of these genetic groups, the impact of recombination on genetic structure, and the potential for maize anthracnose to become a much more significant disease in the future.

Clustering analyses based on single-nucleotide polymorphisms showed strong population differentiation, with the isolates grouped according to their country of origin. The European clade showed less genetic variation, a higher number of repeated MLGs, and lower pairwise differentiation among clusters, suggesting a larger clonal component compared to the other clades. Interestingly, all the shared genotypes were almost genetically identical, suggesting that gene flow is dependent on the clonal isolates generated by asexual reproduction. Most MLGs were shared between isolates from the same cluster and country; however, one MLG encompassed isolates from France and Switzerland (more than 1,000 km apart) and another included isolates from Argentina and Croatia. In our cluster analyses, the European clade encompassed all isolates from Argentina. Together, these results indicate intra- and intercontinental migration of this pathogen. We argue a likely European origin of the isolates from Argentina due to the movement of maize germplasm into South America. Maize breeders in North America and Europe commonly use fields in South American (including Argentina) to advance breeding programs during the Northern Hemisphere winter. Because Argentina is a maize exporter, breeding programs represent a possible source of pathogen importation and consequently a source of pathogen introduction into new areas, for instance, in Europe or elsewhere. This result is particularly important from an epidemiological point of view, with direct implications for disease management because migration can cause the movement of more virulent and/or fungicide resistant genotypes (59).

Contaminated seeds are thought to be the predominant form of dissemination for *C. graminicola* (60–62). Fungi can naturally be spread via the dispersal of both sexual and asexual spores, mainly caused by the transport of infected plant material (11, 56, 63). Dispersal of asexual conidia of *C. graminicola* is strongly dependent on water splash and blowing raindrops, responsible for short-distance dispersal (21). Although airborne dispersal is not typically documented, ascospore dispersal is associated with long-distance dissemination because these spores can be ejected up and dispersed by air currents, traveling longer distances (89, 90). If sexual reproduction occurs even sporadically in this species, it may contribute to long-distance dissemination, leading to a broader pathogen distribution.

The North American clade is more genetically diverse and seems to have a separate evolutionary history. This clade encompassed isolates from the United States and Canada and one strain from Nigeria obtained from a culture collection. Although the first report in the literature of a *Colletotrichum* species infecting maize came from Italy in 1852 (47), we argue that the North American cluster may be the older lineage (or ancestral lineage), even though maize anthracnose was not described in the United States until 1914 (91). Considering that Mesoamerica, most likely Mexico, is the center of origin for maize (92, 93) and that the center of origin of a pathogenic species usually corresponds to that of its host (94) this lineage may be the founder of the 1970s outbreak and may have served as the source for introductions in other continents, given Mesoamerica’s proximity to the USA. The highest genetic diversity found in this lineage also corroborates this hypothesis because a pathogen’s center of origin is likely to consist of populations with more genetic variability than recently founded populations (95). *C. graminicola* may have evolved together with its host in Central America and, due to the expansion of maize production in the USA corn belt, have had an opportunity to spread across the vast maize-growing areas. Alternatively, our sampling, which consisted of a large range of years (1975 to 2016), may have overestimated the genetic diversity of this group. A more contemporary sampling could reveal the current genetic variation of North American populations.

In South America, isolates from Argentina and Brazil were grouped into separate clades, suggesting independent introductions. In Brazil, the first disease report was in 1965 in the state of São Paulo (Campinas) (96). In the country, maize is planted in two distinct growing seasons, with the first crop planted in the summer and the second during the winter. This practice contributes to an increasing primary inoculum in the area. Cota et al. (41) reported a significant reduction in maize yield in tested hybrids which was associated, among other factors, with higher pathogen aggressiveness and greater genetic differentiation in populations during the crop cycle. This could indicate changes in the population size and genetic composition, which may have contributed to a higher disease virulence in the country.

We found molecular evidence of genetic recombination for the three *C. graminicola* populations detected in this study. The rapid decay of linkage disequilibrium and extensive reticulations in the neighbor network is consistent with a possible history of recombination. Furthermore, the PHI test for clonality detected recombination within each population. These results corroborate a previous study that found evidence of recombination using the PHI test at the chromosome level (97). Similar findings of low and fast LD decay have been reported in other fungal plant pathogens (i.e., half its maximum value over a distance of 10 kb or less), suggesting the occurrence of genetic mechanisms responsible for creating such patterns in genomes (78, 98, 99). In addition, the signature of repeat-induced point mutation (RIP), a genome defense mechanism which occurs during sexual reproduction (100, 101), was detected in *C. graminicola* genomes (102, 103). RIP mutations may indicate an ancestral state of sexual reproduction or that meiosis cryptically occurs in nature (67, 104).

Despite *C. graminicola* being reported as a homothallic species, strains may exhibit homothallism and heterothallism, although this phenomenon is not yet fully understood (21). Investigation of fertility through crosses of the strains M1.001 and M5.001 produced exclusively heterothallic offspring, which led Vaillancourt et al. (75) to hypothesize that unbalanced heterothallism could be involved in the mating system. Our investigation of sexual reproduction-related genes revealed gene losses in most isolates and in those species known to be self-fertile, such as *C. salicis*; and those known to be self-sterile and cross-fertile, like *C. fiorinae* (105). Interestingly two genes missing in all *C. graminicola* genomes analyzed were *PPG1* and *PRE2*, which form a pheromone receptor pair. The importance of this signaling pair varies among homothallic ascomycetes. However, none of the deletion mutants obtained in Sordaria macrospora, Aspergillus nidulans, or *Gibberella zeae* completely lost the capability to sexually cross (106–108).

Considering this evidence and the high completeness of the assemblies, we conclude that at least three of the *C. graminicola* isolates (M1.001, LARS318, and CBS113173) maintained all the genes required to undergo sexual reproduction. This finding also suggests that some lineages have lost the ability for sexual reproduction or that not all sexual reproduction-related genes reported in other species are required in *Colletotrichum* spp. (21). Considering the presence of homothallic and heterothallic strains in this species, the lack of genes required to sexually cross in a strain could lead to failure in sexual reproduction. In this scenario, sexual reproduction would become rare (but not inconceivable) due to the low chance of two strains with a full set of genes encountering themselves and mating.

The reproductive biology of the genus *Colletotrichum* is mixed, and the sexual state of several species is unknown, although several species are known to have the capacity for sexual reproduction. The sexual capability of *C. graminicola* is well-documented (74, 109). We experimentally demonstrated the sexual capability of *C. graminicola* isolates to mate under lab conditions, as described by Vaillancourt et al. (75) (see Fig. S4 for details), although the teleomorph has not yet been found in field conditions. Although the absence of the sexual state in nature could lead to less recombination, population genetic studies have shown that several *Colletotrichum* species known to be exclusively asexual exhibit evidence of genetic recombination (78, 79, 110). Some studies have described alternative mechanisms, such as the parasexual cycle, as being responsible for creating genetic recombination in *Colletotrichum* species (75, 80, 111–113).

10.1128/mbio.02878-22.4 FIG S4 Sexual structures of *Colletotrichum graminicola* produced by crossing from strains M1.001 (CB130836) and M5.001 (CBS130839). The cross-inoculation experiment was performed under laboratory conditions as described by Vaillancourt and Hanau 1991 (109). (a) Agar plugs containing strains growing on autoclaved leaves of maize. (b) Mature perithecia. Picture taken using an Olympus SZX9 stereomicroscope outfitted with an Olympus DP70 camera. (c) Ascus and ascospores. (d) Ascospores. Download FIG S4, PDF file, 0.9 MB. Copyright © 2022 Rogério et al. 2022 Rogério et al. https://creativecommons.org/licenses/by/4.0/ This content is distributed under the terms of the Creative Commons Attribution 4.0 International license.

Species with the potential for genetic exchange are more likely to overcome control measures because recombination can result in new combinations of alleles which increase diversity and speed up adaptation at the field scale (59, 114). This study shows evidence that *C. graminicola* populations may have been affected by genetic recombination, but also that, even if it is rare (possibly due to frequent losses of sexual reproduction- and meiosis-associated genes), *C. graminicola* can undergo sexual recombination. Fine-scale investigations at the genome level can be used to determine the real impact of genetic recombination on genetic diversity and adaptation; for instance, whether genetic exchange enhances virulence factors (also called fitness-related traits) in these populations, as has been suggested for other fungal species (115–119). This can be particularly important in the agricultural pathosystem given that crop pathogens constantly face a homogeneous environment which imposes strong directional selection, leading to the evolution of more virulent and drug-resistant populations (94, 120).

We found no correlation between virulence and population structure, although we observed substantial differences in the spectrum of virulence. Several isolates were more aggressive than the strain M1.001, which was originally collected from North America in 1972 during the US outbreak (121). This finding is particularly important because this strain represents the genetic diversity existent in the outbreak and more aggressive strains are currently present in nature, which may present a risk of the development of new, even more destructive outbreaks. A pathogenicity assay was performed in one maize line (Mo940) which was notably susceptible to anthracnose. The use of distinct maize genotypes can result in different responses to fungal infection, as has already been reported in previous studies, and it should be considered in further pathogenic characterizations (40, 122, 123). Another significant aspect is that resistance to maize anthracnose is inherited quantitatively, i.e., host resistance is controlled by several genes with additive genetic effects (124–126). This fact may correlate with the range of virulence found in this study. The three genetic clades of *C. graminicola* detected may have distinct evolutionary origins, encompassing a diverse combination of virulence genes, which could cause different defense responses in the host. We also highlighted that the large variability in virulence should be considered in the screening of resistant maize varieties. Population studies can support breeding programs by investigating the intraspecific variability of *C. graminicola* populations because the effectiveness of genetic resistance depends on knowledge of the genetic structure of pathogens.

### Conclusions.

Our study represents the first investigation of the population structure of *C. graminicola* infecting maize. The three main clades (North America, Brazilian, and European) have different levels of genetic diversity, suggesting that each has a unique evolutionary history. We also found evidence of recent migration of isolates between Europe and Argentina, possibly due to the importation of infected plant material. Although the sexual state of *C. graminicola* has only been reported under lab conditions, we show strong evidence that genetic recombination, whether by sexual reproduction or alternative mechanisms (such as the parasexual cycle), plays an important role in *C. graminicola* population structure. In contrast to the traditional view of *C. graminicola* being mainly asexual, we conclude that genetic recombination is frequent. Fine-scale investigations at the genome level can be employed to understand the real impact of recombination and gene flow on the genetic diversity and adaptation of this species, with a direct effect on control measures and breeding programs aimed at resistance. Finally, we warn that maize anthracnose has the potential to become much more significant in the future, mainly due to isolates which are highly aggressive against the cultivar evaluated, the geographic range expansion of the pathogen, and the increased susceptibility of agroecosystems accentuated by ongoing climate changes.

## MATERIALS AND METHODS

### Sampling and isolation of *C. graminicola*.

Fresh collections of *C. graminicola* samples were obtained from symptomatic maize stems provided by an international network of maize researchers and producers, allowing sampling from nine different countries where anthracnose disease is prevalent (Fig. 1). Eleven *C. graminicola* isolates originally collected from five countries (Germany, Japan, Netherlands, Nigeria, and Zimbabwe) (97, 127) were obtained from public culture collections (Table S1) and included in the collection. Symptomatic stems were dissected, surface-disinfected, and plated in culture medium for fungal isolation, as previously described (20, 54). Fungal structures (hyphal tips, acervuli) emerging from maize tissue were transferred to potato dextrose agar (PDA) medium, allowed to grow for 7 days at 23°C, then single-spore purified. Monosporic cultures were stored at −80°C from suspension conidia in 7.5% skim milk (Merck, Darmstadt, Germany) mixed on silica gel (S/0730/53; Thermo Fisher Scientific, Waltham, MA, USA) (128). Isolates were verified as *C. graminicola* by inspection of colony morphology, spore size, and shape, and by sequencing of the internal transcribed spacer (ITS) region of rDNA using the primers ITS4 and ITS5 (129), following the PCR amplification conditions described by Rech et al. (97). The amplified ITS fragments were purified using NucleoSpin gel and PCR Clean-Up kits (Macherey-Nagel, Düren, Germany) and sequenced using Sanger technology by the University of Salamanca (Spain). Species identity was confirmed by BLAST searches performed against GenBank (NCBI). All materials used in this study were used in accordance with the rights and obligations of the recipient as described in the International Treaty on Plant Genetic Resources for Food and Agriculture adopted by the Food and Agriculture Organization (FAO) on 3 November 2001.

### DNA extraction, library preparation, and sequencing.

Mycelia from monosporic cultures were incubated on an orbital shaker in potato dextrose broth (PDB) (Difco, Becton, Dickinson and Company, Franklin Lakes, NJ, USA) for 3 days at 25°C and 150 rpm under continuous light (52). Genomic DNA was extracted using DNeasy Plant Mini Kits (Qiagen Inc., Valencia, CA, USA) and quantified using a Qubit 3.0 fluorimeter (Invitrogen, Waltham, MA, USA). DNA for *C. graminicola* isolates used for whole-genome sequencing was extracted using the cetyltrimethylammonium-bromide (CTAB) protocol adapted from Baek and Kernerley (130).

New DNA sequence data sets were generated from 97 *C. graminicola* isolates using either RAD-seq or WGS; sequences for an additional 11 isolates were also included in the analyses (97) (Tables S1 and S2). A total of 87 isolates were genotyped with RAD-seq by Floragenex Inc. (Beaverton, OR, USA). Briefly, RAD-seq libraries with sample-specific barcode sequences were constructed from DNA digested with the restriction enzyme PstI, then single-end sequenced (1 × 100 bp) on one lane using a TrueSeq PE150 kit (Illumina, Inc., San Diego, CA, USA) on an Illumina HiSeq 2000 instrument. WGS data were obtained from seven *C. graminicola* isolates (CBS252.59, F-64330-2, F-64330-7, F-40330-1, SW-8046-2, CRO-I-35, CR-10370-50) from libraries prepared using a Nextera XT DNA kit (Illumina, Inc.) and sequenced on an Illumina HiSeq 4000 (2 × 150 bp). WGS data were obtained from six *C. graminicola* isolates (ARG-2700-2, CA-CHAT-1, CA-N8H, CRO-I-41, P-7565-072-1, SW-8046-1) from paired-end libraries prepared using an Illumina TruSeq DNA Nano kit and sequenced on an Illumina MiSeq (2 × 300 bp). Sequence quality was checked on FastQC v0.11.7 (Babraham Bioinformatics, Cambridge, United Kingdom) and low-quality reads and adaptors were trimmed with Trimmomatic v0.3.8 (131) using the default settings. *De novo* assemblies were performed with SPAdes v3.11.1 (132). Low-coverage scaffolds were identified and those with coverage of less than 10% of the nuclear genome (based on the coverage of the largest contig) were removed. The contigs corresponding to the mitochondrial genome were identified by local BLASTn using the *C. graminicola* mitochondrial sequence (accession no. CM001021.1) as the query sequence. To identify the rRNA gene clusters, we used the following query sequences: XR_001139483 (28S, GLRG_12546); XR_001139484.1 (18S); and DQ003110.1 (18S rRNA gene, partial sequence; internal transcribed spacer 1, 5.8S rRNA gene, and internal transcribed spacer 2, complete sequence; and 28S rRNA gene, partial sequence). Matching contigs were also removed. The assembly completeness was assessed using Busco v.3 (133) and the sordariomyceta_odb9 lineage data set.

10.1128/mbio.02878-22.6 TABLE S2 Summary statistics of *Colletotrichum graminicola* genomes generated in this study. Download Table S2, XLSX file, 0.1 MB. Copyright © 2022 Rogério et al. 2022 Rogério et al. https://creativecommons.org/licenses/by/4.0/ This content is distributed under the terms of the Creative Commons Attribution 4.0 International license.

### Read mapping and SNP calling.

The RAD-seq reads were inspected with FastQC v0.11.7 (Babraham Bioinformatics, Cambridge, UK) to confirm quality and were demultiplexed and quality-filtered with the process_radtags program in Stacks v.2.10 (134). The demultiplexed reads were trimmed using Trimmomatic v0.3.8 (131) using the parameters ‘leading:15 trailing:15 slidingwindow:4:15 minlen:36’ and aligned to the *C. graminicola* reference genome M1.001 (NCBI accession no. SAMN02953757) using BWA-MEM v0.7.8 (135) with the default settings. Genetic variants were identified with FreeBayes v0.9.21 (136) using the default parameters. The WGS reads were quality-trimmed with Trimmomatic v0.3.8 and aligned to the reference genome with Speedseq v0.1.2 (137), which uses BWA-MEM v0.7.8 (135) for read mapping and FreeBayes v0.9.21 (136) for variant identification. SNPs of high confidence were filtered using VCFtools v0.1.15 (138) with the parameters ‘–max-alleles 2 -min-meanDP 30 -remove-indels -maf 0.03 -max-missing 1.’ Only biallelic SNPs present in all isolates and sites with a minor allele frequency of less than 3% were kept in the data set.

### Population structure and genetic diversity.

We defined a population as a group of individuals of the same species which occupy the same area, colonize the same type of host simultaneously, and share a common evolutionary history (59). Analysis of molecular variance was performed with the hypothesis of no genetic differentiation using the package Poppr v2.8.6 (139). Weir and Cockerham’s genetic differentiation index (*F*_ST_) was calculated with the R package hierfstat v0.5-11 using the WC84 method (140). We used three clustering methods to examine the *C. graminicola* population structure. First, we used a principal-component analysis with *a priori* geographical knowledge from sampling (by continent). This analysis was conducted in R with the package adegenet v2.0.1 (141) using the dudi.pca function. Second, we performed a discriminate analysis of principal components with adegenet v2.0.1 (141). The find.cluster function was used to identify the most likely grouping in the data based on the Bayesian information criterion calculated for 1 to 10 clusters. Third, to confirm the pattern of population subdivision inferred by the PCA and DAPC, we used the fast maximum-likelihood genetic clustering method snapclust (142) in adegenet v2.0.1 (141). The function snapclust.choosek was used as an initial step to determine the optimal number of clusters (*K*) based on the BIC. Additionally, we implemented a neighbor network using the software SplitsTree v4 (143) to visualize phylogenetic signals, indicated by the presence of reticulations in the network, from a distance matrix with default parameters.

The number of multilocus genotypes (MLGs) was calculated using the R package Poppr v2.8.6 (139). We used the mgl.filter function to determine the true number of MLGs from a genetic distance obtained by the diss.dist function using the threshold determined in the cutoff_predictor tool. To visualize relationships among genotypes, an MSN was generated using the *msn* function in Poppr. Genetic diversity was estimated by comparison of the number of SNP variations by counting the number of segregating sites between individuals, using the find.variations tool in Geneious (144).

### Linkage disequilibrium.

The coefficient of linkage disequilibrium (*r*^2^) (145) was calculated using VCFtools (138). The rate of decay in linkage disequilibrium with physical distance was calculated for all pairs of SNPs less than 100 kb apart using the option -hap-r2 and excluding sites with minor allele frequencies below 5%. We also used the pairwise homoplasy index (PHI) test implemented in SplitsTree v4 (143) to test the null hypothesis of clonality.

### Investigation of putative sexual reproduction-related genes.

To identify orthologues of sexual reproduction-related genes in the *C. graminicola* genomes, we used a set of 96 genes with known roles in sexual reproduction in fungal model systems (87, 88). First, we performed BLASTp searches (E value cutoff = 1e −3; 80% amino acid sequence similarity) in the proteome of the reference strain (M1.001) (121) to identify the loci encoding the sexual reproduction-related genes which were investigated to be used as query sequences. The coding sequences retrieved from the identified loci were then used as queries for BLASTn (E value cutoff = 1e −3) on the *C. graminicola* assemblies. The sequences obtained were aligned to the queries and manually checked for interruption in the translation frame. Finally, we assessed the presence/absence of these genes in genomes. For this analysis, we used the 13 *C. graminicola* assemblies generated in this study and the 5 *C. graminicola* genome assemblies used by Rech et al. (97) (Tables S1 and S2) (the isolates JAB2 and MAFF511343 were not used for this analysis due to the low coverage of sequencing). In addition, genome assemblies from nine other fungal species were included in the analysis: *Colletotrichum fiorinae* (genome accession no. JARH00000000.1 [146]), *C. fructicola* (accession no. ANPB00000000.2 [147]), *C. gloeosporioides* (accession no. AMYD00000000.1 [148]), *C. higginsianum* (accession no. LTAN00000000.1 [149]), *C. nymphaeae* (accession no. JEMN00000000.1 [105]), *C. salicis* (accession no. JFFI00000000.1 [105]), *C. simmondsii* (accession no. JFBX00000000.1 [105]), *C. sublineola* (accession no. JMSE00000000.1 [150]), *C. orbiculare* (accession no. AMCV00000000.2 [147]), and Verticillium dahliae (accession no. ABJE00000000.1 [151]). For all species other than *C. graminicola*, BLASTp searches were performed using the proteins encoded by the 96 known sexual reproduction-related genes as query sequences. BLAST results were inspected manually to determine the presence or absence of orthologues.

### Pathogenicity assays.

Pathogenicity assays were performed by *in vivo* leaf blight assays using a derivative of the susceptible maize inbred line Mo940 (152). Plants were cultured in a greenhouse for 2 weeks (V3 developmental stage) and inoculated via *C. graminicola* spore suspensions (3 ×10^5^ spores ml^−1^) according to the methods of Vargas et al. (153). Virulence was quantified by measuring the area of necrotic lesions on leaves 4 days after inoculation. Symptomatic leaves were excised from the plants, scanned with a flat-bed scanner, and quantified using the imaging processing software Paint.NET v4.2.16 (dotPDN LCC, Seattle, WA, USA) (52), with three biological replicates per isolate. An ANOVA with Bonferroni corrections for pairwise comparisons was performed to compare differences in virulence among isolates. Twenty-one isolates were assessed, divided into batches because the pathogenicity assays could not accommodate all isolates simultaneously. Given the possibility of a batch effect, one of the strains (M1.001) was analyzed in all batches to estimate the possible systematic differences among them. We compared the isolate repeated in the different batches using ANOVA with lesion size as the response variable, batch as the predictor variable, and a null hypothesis of no difference between batches. We obtained highly significant differences (*P* < 0.0001), indicating a clear batch effect. Therefore, to eliminate the systematic differences among batches, we standardized each strain using the mean and standard deviation of the control (strain M1.001) for its batch. An ANOVA of the corrected data was used to determine the presence of significant differences among isolates. The second test was to determine whether there were differences among isolates, after correcting the lesion sizes to account for batch differences. The predictor variable was isolate, the response variable was the corrected lesion size, and the null hypothesis was no difference in lesion size among the isolates. The ANOVA test was significant (*P* < 0.0001), so we performed a Tukey’s comparison-of-means test.

### Data availability.

The genome assemblies and raw Illumina RAD-seq reads were submitted to GenBank and their accession numbers are listed in Table S2.
