# Supplementary material for: Population Genomics Provide Insights into the Global Genetic Structure of Colletotrichum graminicola, the Causal Agent of Maize Anthracnose
Source: mBio. 2022 Dec 19;14(1):e02878-22. doi: 10.1128/mbio.02878-22 (PMC9973043; doi:10.1128/mbio.02878-22)
Supplement: FIG S1 [file mbio.02878-22-sf001.pdf]

A)

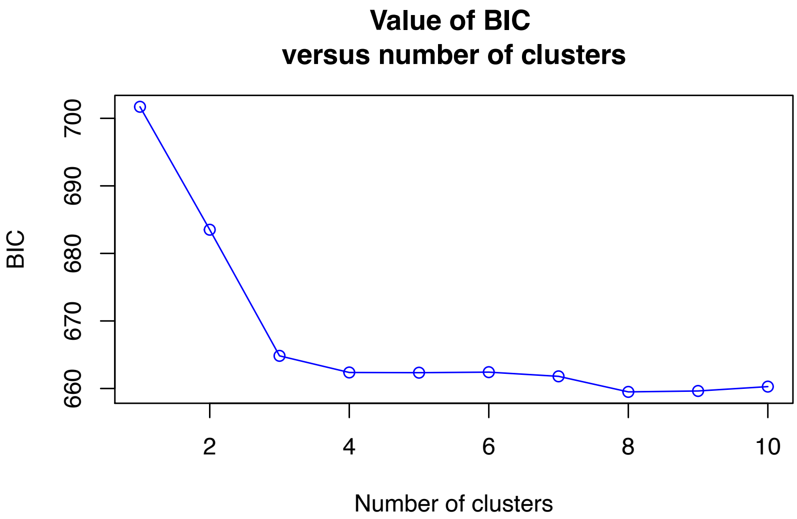

B)

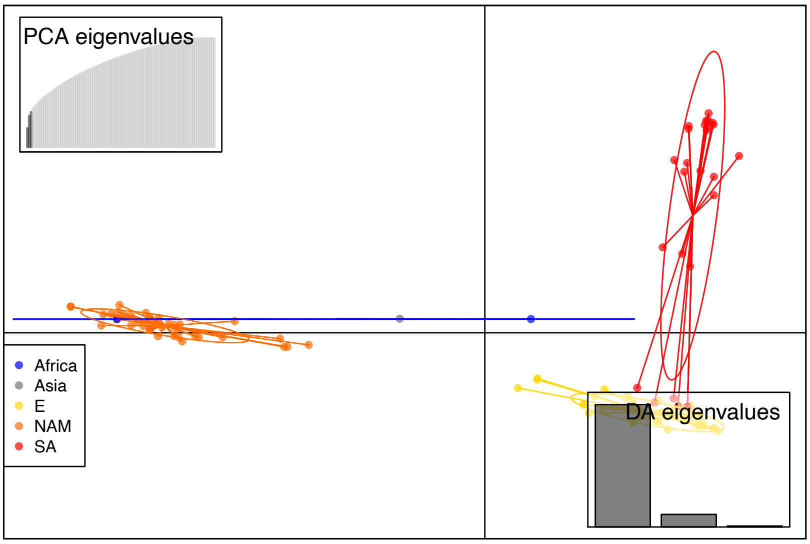

Supplementary Fig. S1. A) Bayesian information criteria (BIC) indicating the most probable number of genetic groups by discriminant analysis of principal components analysis (DAPC). B) Scatterplot from DAPC of *Colletotrichum graminicola* isolates assigned with *a priori* geographical information (by continent).
