# Supplementary material for: Population Genomics Provide Insights into the Global Genetic Structure of Colletotrichum graminicola, the Causal Agent of Maize Anthracnose
Source: mBio. 2022 Dec 19;14(1):e02878-22. doi: 10.1128/mbio.02878-22 (PMC9973043; doi:10.1128/mbio.02878-22)
Supplement: FIG S4 [file mbio.02878-22-sf004.pdf]

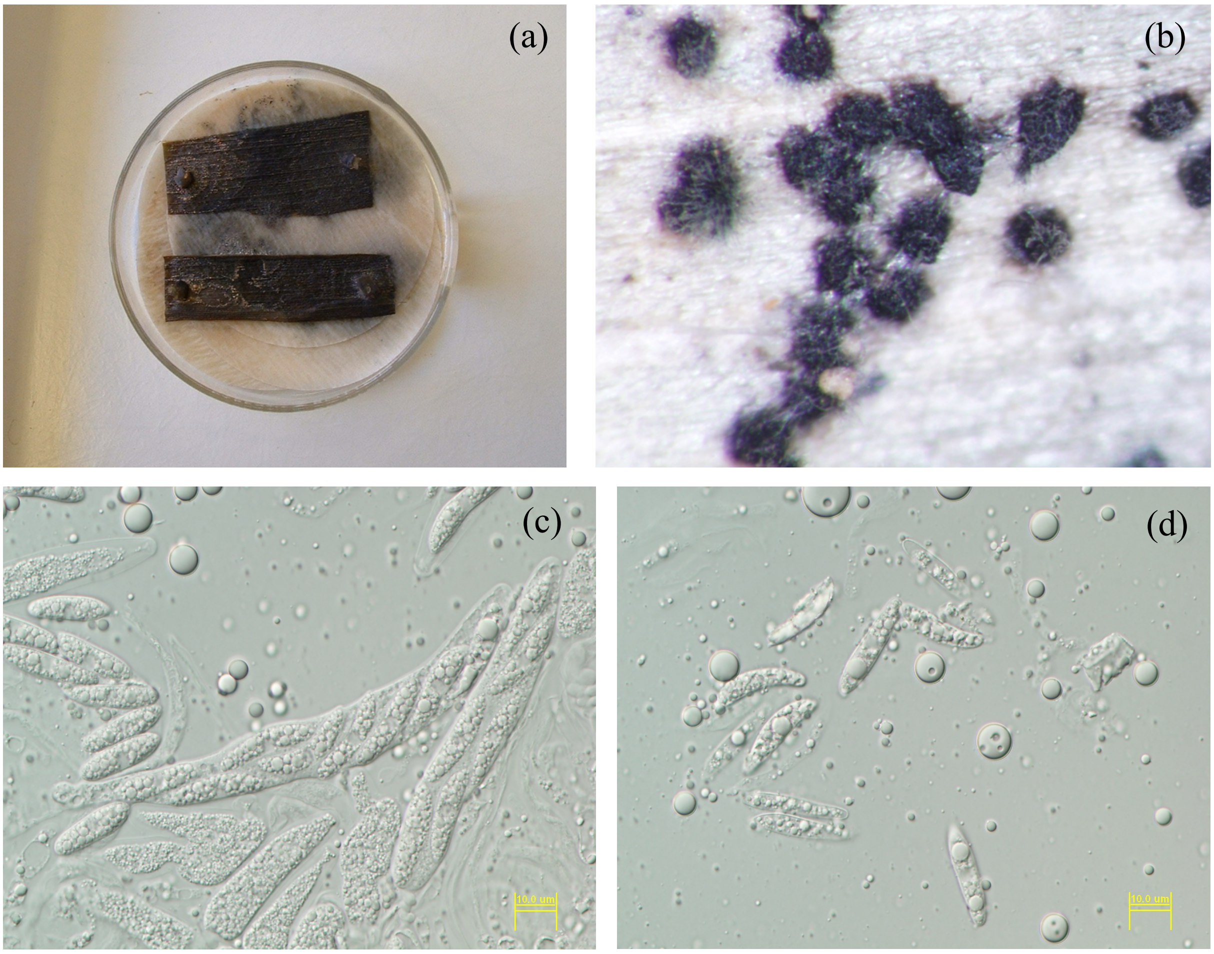

Supplementary Fig. S4. Sexual structures of *Colletotrichum graminicola* produced by crossing from strains M1.001(CB130836) and M5.001 (CBS 130839). The cross-inoculation experiment was performed under laboratory conditions as described by Vaillancourt and Hanau 1991. (a) Agar plugs containing strains growing on autoclaved leaves of maize. (b) Mature perithecia, picture taken with Olympus SZX9 stereomicroscope outfitted with an Olympus DP70 camera. c) Ascus and ascospores. d) Ascospores
